# Impact of possible tardive dyskinesia on physical wellness and social functioning: results from the real-world RE-KINECT study

**DOI:** 10.1186/s41687-023-00551-5

**Published:** 2023-03-09

**Authors:** Caroline M. Tanner, Stanley N. Caroff, Andrew J. Cutler, William R. Lenderking, Huda Shalhoub, Véronique Pagé, Ericha G. Franey, Michael Serbin, Chuck Yonan

**Affiliations:** 1 Weill Institute for Neurosciences, Department of Neurology, University of California - San Francisco, 1651 4th Street, San Francisco, CA 94158 USA; 2 Parkinson’s Disease Research, Education and Clinical Center, San Francisco Veterans Affairs Health Care System, 4150 Clement St., San Francisco, CA 94121 USA; 3 Corporal Michael J. Crescenz Veterans Affairs Medical Center, 3900 Woodland Ave., Philadelphia, PA 19104 USA; 4 Perelman School of Medicine, University of Pennsylvania, 3400 Civic Center Blvd., Philadelphia, PA 19104 USA; 5 SUNY Upstate Medical University, 8429 Lorraine Rd., Lakewood Ranch, FL 34202 USA; 6 Evidera, 500 Totten Pond Rd., Waltham, MA 02451 USA; 7 Evidera, 7575 Trans-Canada Hwy., St-Laurent, QC H4T 1V6 Canada; 8 Neurocrine Biosciences, Inc., 12780 El Camino Real, San Diego, CA 92130 USA

**Keywords:** Tardive dyskinesia, Quality of life, Function, Real-world evidence, Antipsychotic agents

## Abstract

**Background:**

Tardive dyskinesia (TD) is a persistent and potentially disabling movement disorder associated with antipsychotic use. Data from RE-KINECT, a real-world study of antipsychotic-treated outpatients, were analyzed to assess the effects of possible TD on patient health and social functioning.

**Methods:**

Analyses were conducted in Cohort 1 (patients with no abnormal involuntary movements) and Cohort 2 (patients with possible TD per clinician judgment). Assessments included: EuroQoL’s EQ-5D-5L utility (health); Sheehan Disability Scale (SDS) total score (social functioning); patient- and clinician-rated severity of possible TD (“none”, “some”, “a lot”); and patient-rated impact of possible TD (“none”, “some”, “a lot”). Regression models were used to analyze the following: associations between higher (worse) severity/impact scores and lower (worse) EQ-5D-5L utility (indicated by negative regression coefficients); and associations between higher (worse) severity/impact scores and higher (worse) SDS total score (indicated by positive regression coefficients).

**Results:**

In Cohort 2 patients who were aware of their abnormal movements, patient-rated TD impact was highly and significantly associated with EQ-5D-5L utility (regression coefficient: − 0.023, *P* < 0.001) and SDS total score (1.027, *P* < 0.001). Patient-rated severity was also significantly associated with EQ-5D-5L utility (− 0.028, *P* < 0.05). Clinician-rated severity was moderately associated with both EQ-5D-5L and SDS, but these associations were not statistically significant.

**Conclusions:**

Patients were consistent in evaluating the impacts of possible TD on their lives, whether based on subjective ratings (“none”, “some”, “a lot”) or standardized instruments (EQ-5D-5L, SDS). Clinician-rated severity of TD may not always correlate with patient perceptions of the significance of TD.

**Supplementary Information:**

The online version contains supplementary material available at 10.1186/s41687-023-00551-5.

## Background

Tardive dyskinesia (TD) is a persistent and potentially disabling movement disorder that is associated with exposure to antipsychotics and other dopamine receptor blocking agents [1, 2]. While second-generation (“atypical”) antipsychotics have been associated with a lower incidence of TD, prevalence studies have shown the continued importance of TD as a clinical consideration with these therapies due to their expanding use, especially in non-psychotic conditions [3, 4]. Therefore, it is important that all patients taking any antipsychotic medication be screened regularly for potential TD symptoms, including abnormal involuntary movements in the face/mouth, neck/trunk, upper extremities, and lower extremities [5].

Early case reports of TD suggested that it may be reversible if diagnosed early in some patients who could be safely withdrawn from antipsychotics and followed over time; therefore, when appropriate, tapering off antipsychotic medications could be tried when TD first emerges [6]. However, antipsychotic discontinuation is not a practical option for patients with chronic psychotic disorders, and the overall evidence for discontinuation leading to resolution of TD is insufficient, especially in established cases [7]. In these cases, use of an approved TD medication (i.e., vesicular monoamine transporter 2 inhibitor) is appropriate. Moreover, current treatment guidelines indicate that factors such as patient preference, impairment, or psychosocial functioning should be considered when planning treatment [8]. Consistent with these recommendations, results from a modified Delphi consensus study indicated that improvements in patient-reported distress, functional impairments, and health-related quality of life (HRQoL) should be considered when determining treatment success in patients with TD [9].

The impact of TD can be wide-ranging. The cumulative effects of TD on patients with a serious mental illness may include worsened psychopathology, higher rates of comorbidities, increased risk of mortality, and poorer treatment outcomes [10–12]. TD can negatively affect motor functions such as speech, gait, and respiration, as well as cognitive functions such as verbal memory and processing [11, 13, 14]. TD can also lead to feelings of stigmatization, social withdrawal, loss of employment, and higher healthcare resource utilization [12, 15, 16].

Although many clinicians, patients, and caregivers are aware of these negative impacts, quantitative assessments of HRQoL in TD are very limited. RE-KINECT was a real-world study that included psychiatric outpatients who had been treated with antipsychotics. Previously published results from this study indicated that 27.6% of these patients had abnormal involuntary movements that were consistent with possible TD, and that the impact of these movements on both patients and caregivers was considerable [17, 18]. Relevant to the current analyses, RE-KINECT included two established patient-reported HRQoL measures: EuroQoL’s 5-dimension 5-level questionnaire (EQ-5D-5L) and the Sheehan Disability Scale (SDS). Analyses based on these outcomes were used to further explore the impact of TD on patients’ overall health, physical wellness, and social functioning. The findings from these analyses are intended to provide the type of information needed for appropriate health technology assessments, as needed for the evaluation of medications used to treat TD. In a broader sense, the quantitative analyses in this report are useful for understanding the impact of TD beyond subjective impressions. Moreover, they allow for comparisons among different types of patients (e.g., those with no abnormal involuntary movements versus those with possible TD).

## Methods

### Study design

RE-KINECT was a prospective, real-world, observational, multicenter study conducted at 37 outpatient psychiatry clinics (e.g., research institutions, community health centers, private practices) in the United States from April 2017 to January 2018 [17]. In brief, this study included adults with ≥ 3 months of lifetime exposure to antipsychotic medication and ≥ 1 clinician-confirmed psychiatric disorder according to *Diagnostic and Statistical Manual of Mental Disorders, Fifth Edition* (DSM-5) criteria [19]. The 3 month lifetime antipsychotic exposure requirement was based on the Schooler-Kane criteria for TD research and DSM-IV-TR [20, 21], and it is consistent with current American Psychiatric Association guidelines (DSM-5-TR) [22]. All patients provided written informed consent prior to participation; Institutional Review Board approval was obtained at each site.

### Patient cohorts

**Fig. 1:**
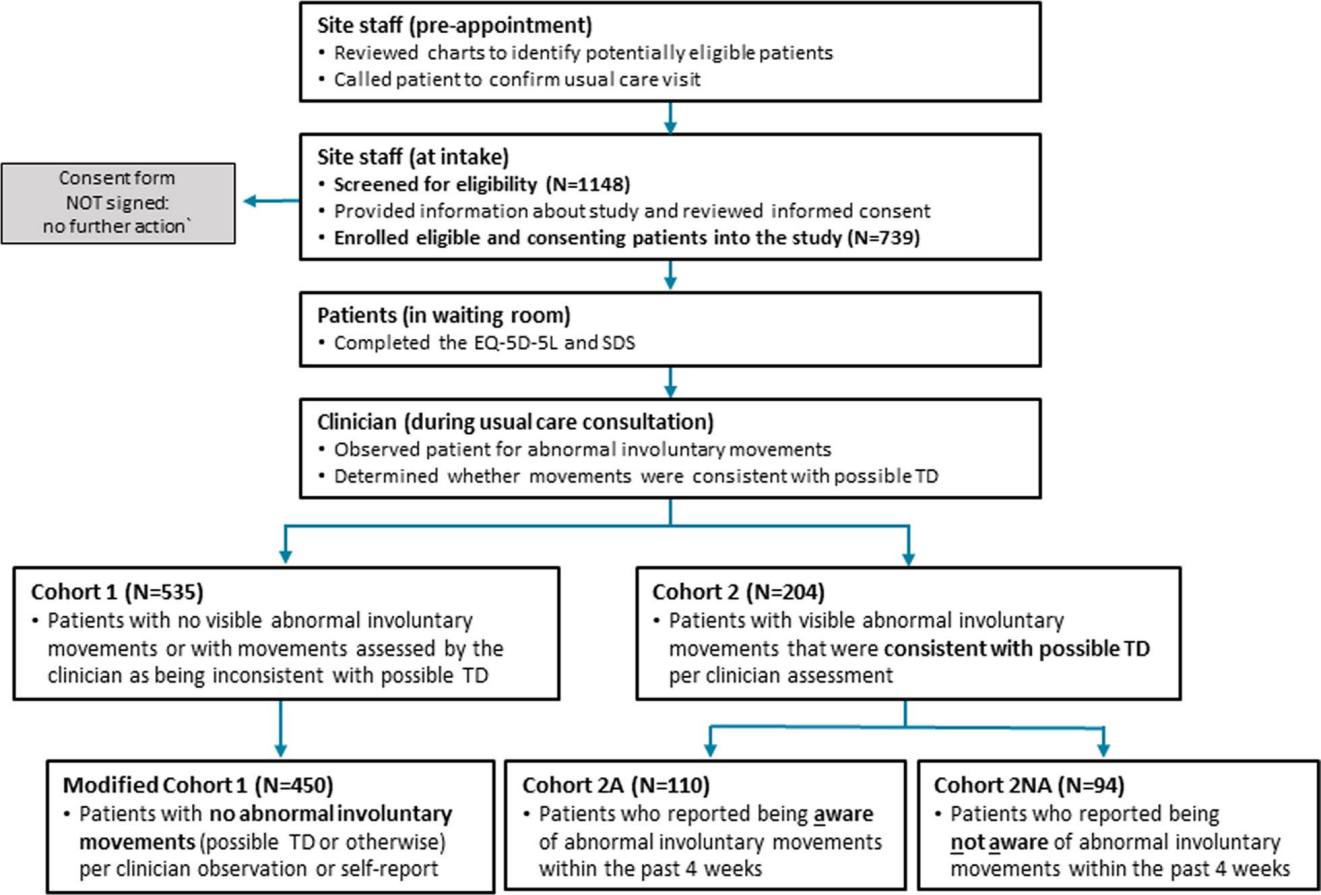
Overview of possible TD symptom screen and cohort assignment. EQ-5D-5L, EuroQoL 5-dimension 5-level questionnaire; SDS, Sheehan Disability Scale; TD, tardive dyskinesia

Patients were assigned to 1 of 2 cohorts based on clinician assessment (Fig. 1). Training for TD screening (via videos) was provided to all clinical site personnel to promote inter-rater reliability. Cohort 1 was defined in the study protocol as patients who had no abnormal involuntary movements or whose movements were not consistent with possible TD based on clinician assessment. To avoid any potential overlap with patients with possible TD (as described below), this analysis focused on a modified Cohort 1 (no abnormal involuntary movements), which excluded patients with non-TD involuntary movements such as tremor.

Cohort 2 was defined in the study protocol as patients who had abnormal involuntary movements that were confirmed by their clinician as possible TD. All patients in Cohort 2 had a clinician-rated severity of possible TD as “some” or “a lot” in at least 1 of the following body regions: head/face, neck/trunk, upper extremities, and/or lower extremities. For this analysis, Cohort 2A (“**a**ware”) was defined as patients with clinician-confirmed possible TD who also self-reported having abnormal involuntary movements within the past 4 weeks and had a self-rated severity of “some” or “a lot” in at least 1 of the 4 body regions. Data from Cohort 2NA (“**n**ot **a**ware”), defined as patients with possible TD who self-reported having no abnormal involuntary movements in the past 4 weeks, were analyzed to provide supplementary information about the potential effects of patient awareness on HRQoL.

### Assessments

Prior to clinician assessment and cohort assignment, all eligible and consenting patients were asked to complete the EQ-5D-5L [23], which assesses problems in 5-dimensions (mobility, self-care, usual activities, pain/discomfort, anxiety/depression) that can be used to calculate a utility score, which ranges from 0 (health state equivalent to death) to 1 (perfect health). The EQ-5D-5L also includes a visual analog scale (VAS) that measures current health state, which ranges from 0 to 100 (best possible health). Patients were also asked to complete the SDS [24], which assesses disruption in 3 social functioning domains (work/school, social life, family life/home responsibilities) and includes a total score (sum of domain scores) which ranges from 0 (no disruption) to 30 (extreme disruption).

For severity of possible TD, clinicians and patients were asked to “rate the severity of visible, uncontrollable movements” for each of 4 body regions (head/face, neck/trunk, upper extremities, and/or lower extremities) using simple descriptors of “none”, “some”, or “a lot”. For impact of possible TD, patients who were aware of their abnormal involuntary movements (Cohort 2A) were asked to rate how much “over the past 4 weeks” did these movements “impact your ability” to perform each of 7 different activities/functions (usual activities, talking, eating, breathing, being productive, self-care, socializing), also using the descriptors of “none”, “some”, or “a lot”. For regression analyses, these descriptors were assigned values of 0, 1, and 2, respectively, with the summary score for severity ranging from 0 (“none” in all 4 regions) to 8 (“a lot” in all 4 regions) and the summary score for impact ranging from 0 (“none” in all 7 activities) to 14 (“a lot” in all 7 activities).

### Analyses

All analyses were intended to be exploratory, and no adjustments were made for multiple comparisons. Patients’ demographics, clinical characteristics, EQ-5D-5L scores, and SDS scores were analyzed for Cohorts 2 and 2A versus modified Cohort 1, as well as for Cohort 2A versus Cohort 2NA. Chi-squared tests were used for categorical variables and t-tests were used for continuous variables. Analyses were adjusted for potentially confounding factors as follows: age (18–95 years); sex (male or female); patient-reported overall health status (0 = “no health problems” to 10 = “health as bad as you can imagine”); clinician-rated severity of psychiatric condition (0 = “normal, not ill” to 6 = “among the most severely ill”); clinician-rated functional status of patient (0 = working/studying/managing household independently to 2 = not working/studying/managing household in any capacity); and psychiatric diagnosis (presence or absence of schizophrenia or schizoaffective disorder, presence or absence of a mood or other disorder). These factors were based on clinically relevant characteristics that would likely affect HRQoL in patients with TD; no statistical process was used for selection.

Simple linear regressions were used to evaluate EQ-5D-5L utility and SDS total scores by clinician/patient-rated severity of possible TD and by patient-rated impact of possible TD. These analyses compared Cohorts 2 and 2A to modified Cohort 1, based on mean score differences between the cohorts. Simple linear regressions were also used to determine the associations between possible TD (clinician/patient-rated severity, patient-rated impact) and HRQoL measures—physical wellness (EQ-5D-5L utility) or social function (SDS total) in Cohort 2, Cohort 2A, and Cohort 2NA. These analyses were adjusted for age, sex, patient-reported overall health status, clinician-rated severity of psychiatric condition, clinician-rated functional status of patient, and psychiatric diagnosis.

## Results

### Patient cohorts and baseline characteristics

A total of 1148 patients from 37 clinical sites in 19 states were screened, including patients from research institutions, community health centers, and private psychiatric practices. Modified Cohort 1 included 450 patients who had no abnormal involuntary movements (possible TD or otherwise) (Fig. 1). The full Cohort 2 included 204 patients who had abnormal involuntary movements that were clinician-confirmed as possible TD, with a clinician-rated severity of “some” or “a lot” in ≥ 1 body region. Cohort 2A (aware) included 110 patients with self-reported abnormal involuntary movements in the past 4 weeks, with a self-rated severity of “some” or “a lot” in ≥ 1 body region. Cohort 2NA (not aware) included 94 patients who had clinician-confirmed possible TD but self-reported no abnormal involuntary movements within the past 4 weeks.

Compared to patients with no abnormal involuntary movements (modified Cohort 1), patients with possible TD (Cohort 2) were significantly older, with a higher proportion of male patients, higher incidence of schizophrenia or schizoaffective disorder, and lower incidence of mood or other psychiatric disorders (Table 1). Per clinician impression, patients with possible TD also had more severe psychiatric conditions and were less likely to be engaged in what many would consider “usual activities” such as working/studying or managing a household independently and more likely to not engage in these activities at all. Per patient self-report, those with possible TD had worse overall health status than those with no abnormal involuntary movements.

**Table 1:** Demographics and clinical characteristics

	Modified Cohort 1 (N = 450)^a^	Cohort 2 (N = 204)^a^	*P*-value^b^
Age, mean (SD)	47.5 (14.6)	54.6 (13.6)	< 0.001
*Sex, n (%)*			
Male	182 (40.5)	100 (49.0)	0.043
Female	267 (59.5)	104 (51.0)	
Lifetime antipsychotic exposure, mean (SD), years	9.5 (9.0)	15.9 (13.9)	< 0.0001
*Psychiatric condition, n (%)*^*c*^			
Schizophrenia or schizoaffective disorder	146 (32.4)	107 (52.5)	< 0.001
Mood or other psychiatric disorder	354 (78.7)	134 (65.7)	< 0.001
*Severity of psychiatric condition, n (%)*			
Normal, not ill	47 (10.4)	7 (3.4)	< 0.001
Minimally ill	103 (22.9)	27 (13.2)	
Mildly ill	111 (24.7)	68 (33.3)	
Moderately ill	128 (28.4)	67 (32.8)	
Markedly ill	43 (9.6)	26 (12.7)	
Severely ill	16 (3.6)	9 (4.4)	
Among the most severely ill	2 (0.4)	0	
Overall health status, mean (SD)^d^	4.2 (2.8)	4.7 (2.8)	0.031
*Overall functional status, n (%)*^*e*^			
Working/studying independently	242 (53.8)	66 (32.4)	< 0.001
Working/studying with assistance	100 (22.2)	44 (21.6)	
Not working/studying	108 (24.0)	94 (46.1)	

*SD* standard deviation; *TD* tardive dyskinesia

^a^Modified Cohort 1 includes patients with no visible or self-reported abnormal involuntary movements. Cohort 2 includes all patients with possible TD per clinician assessment

^b^For questions or items that allowed more than 1 response (i.e., categories not mutually exclusive), *P*-values are provided for each response. Chi-squared tests were used for categorical variables; t-tests were used for continuous variables

^c^Based on questionnaire responses (i.e., not diagnostic medical codes). Mood or other psychiatric disorders include bipolar disorder, major depressive disorder, anxiety disorder or symptoms, post-traumatic stress disorder, personality disorder, attention deficit hyperactivity disorder, substance use disorder, and other psychotic disorder

^d^Per patient self-report. Higher scores indicate worse overall health (range, 0 = “no health problems” to 10 = “health as bad as you can imagine”)

^e^Per clinician impression, based on the following options: (1) been independently working or studying full- or near full-time, in usual occupation; or managing own household; or participating in unpaid or voluntary activities, whether retired or not; (2) been working or studying with assistance in usual occupation and/or managing own household or participating in unpaid or voluntary activities; or has experienced a significant reduction in house work; or was in a sheltered situation or on sick leave; and (3) not been working or studying in any capacity and not managing own household

No significant differences between Cohort 2NA and Cohort 2A were found for age, sex, psychiatric condition, clinician-rated severity of psychiatric condition, or clinician impression of overall functional status. However, compared to Cohort 2NA, patients in Cohort 2A had significantly worse self-reported overall health (Additional file 1: Appendix Table S1).

### EQ-5D-5L and SDS scores

When adjusted for age, sex, health status, severity of psychiatric condition, functional status, and psychiatric diagnosis, mean baseline EQ-5D-5L scores were lower in patients with possible TD ﻿(Cohort 2) than in patients with no abnormal involuntary movements (modified Cohort 1), indicating relatively worse overall health (utility score and visual analog scale [VAS]) (Table 2). Mean SDS dimension and total scores were higher in Cohort 2 compared to modified Cohort 1, indicating relatively worse social functional status in patients with possible TD than in patients with no abnormal involuntary movements; however, none of the differences in mean scores were statistically significant. Differences between Cohorts 2A and 2NA for mean SDS total and family/home life scores indicated statistically significantly worse social functioning in patients who were aware of their possible TD than in those who were not aware (Additional file 1: Appendix Table S2).

**Table 2:** Mean EQ-5D-5L and SDS scores

	Modified Cohort 1^a^		Cohort 2^a^		*P-*value^b^
	n	Mean (SD)	n	Mean (SD)	
*EQ-5D-5L scores*^*c*^					
Health state VAS	446	70.4 (21.4)	204	66.8 (25.1)	0.2068
Utility score	442	0.78 (0.18)	197	0.71 (0.21)	0.0163
*SDS scores*^*d*^					
Work/school	310	3.5 (3.4)	111	4.2 (3.4)	0.2559
Social life	446	3.5 (3.2)	203	4.0 (3.4)	0.8237
Family/home life	445	3.4 (3.2)	203	3.8 (3.3)	0.8449
Total score	445	10.5 (8.8)	203	11.7 (9.3)	0.7245

*EQ-5D-5L* EuroQoL 5-dimension 5-level questionnaire; *SD* standard deviation; *SDS* Sheehan Disability Scale; *VAS* visual analog scale

^a^Modified Cohort 1 includes patients with no visible or self-reported abnormal involuntary movements. Cohort 2 includes all patients with possible TD per clinician assessment

^b^Adjusted for age, sex, overall health status, severity of psychiatric condition per clinician impression, functional status of patient per clinician impression, and psychiatric diagnosis

^c^Higher EQ-5D-5L scores indicate better health-related quality of life: VAS (range, 0 = “worst health you can imagine” to 100 = “best health you can imagine”); utility (range, 0 = “health state equivalent to death” to 1 = “perfect health”)

^d^Higher SDS scores indicate greater disruption due to health condition: domain scores (range, 0 = “not at all” [no disruption to work/school, social life, or family/home life] to 10 = “extremely” [extreme disruption]). Total scores (range, 0–30) were calculated for patients who had a score on ≥ 2 domains. When only 1 domain was missing, the average of the patient’s observed score was imputed

**Fig. 2:**
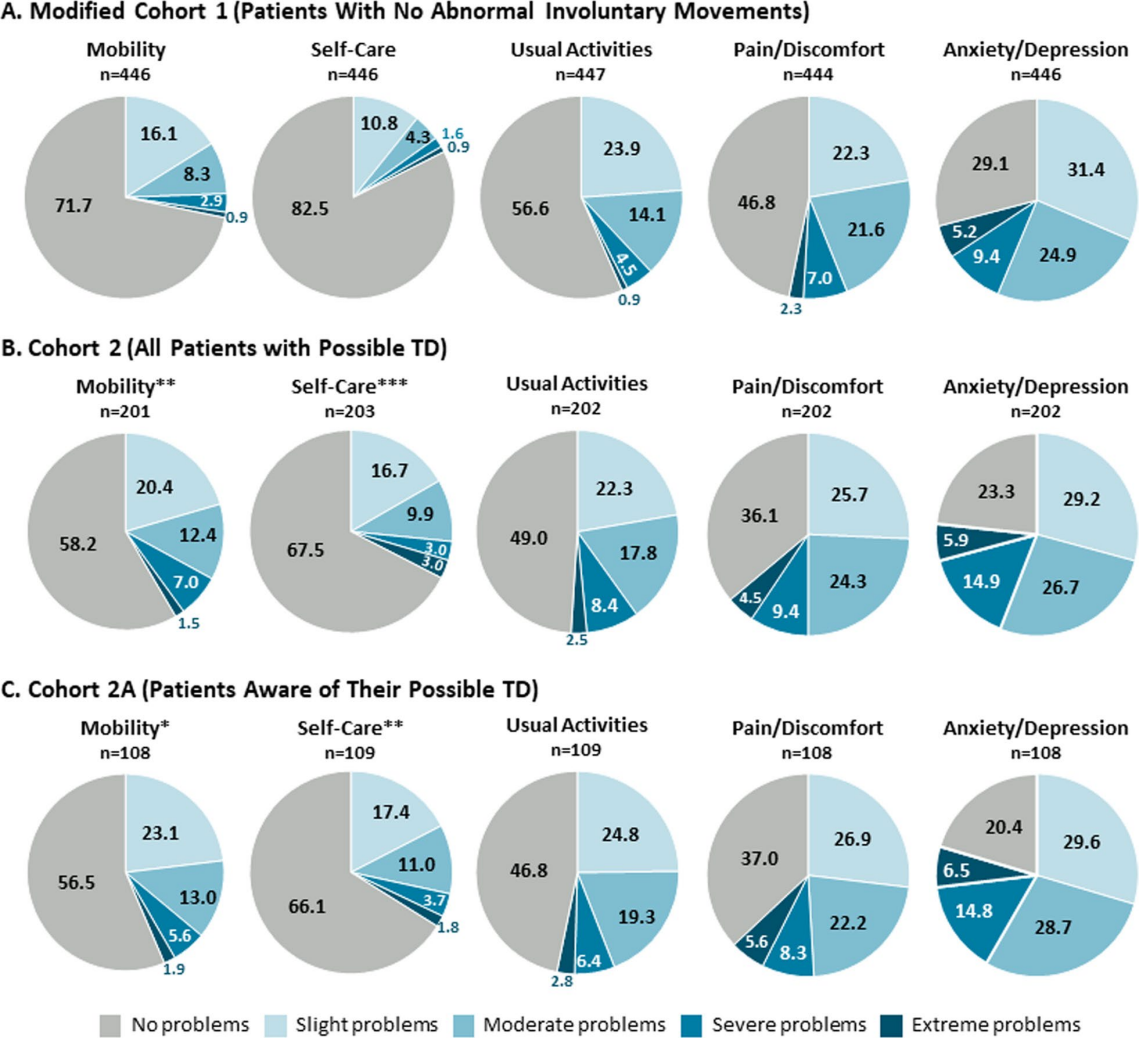
Distribution of EQ-5D-5L scores. *P < 0.05; **P < 0.01; ***P < 0.001 versus modified Cohort 1. EQ-5D-5L, EuroQoL 5-Dimension 5-Level questionnaire; TD, tardive dyskinesia

Compared to patients with no abnormal involuntary movements (modified Cohort 1), a higher percentage of patients with clinician-confirmed possible TD (Cohorts 2 and 2A) reported having moderate problems (score = 3), severe problems (score = 4), or extreme problems (score = 5) in all EQ-5D-5L dimensions (Fig. 2). Interestingly, the dimensions of mobility and self-care were significantly worse in Cohorts 2 and 2A than in modified Cohort 1. Compared to patients who were aware of their possible TD (Cohort 2A), significantly fewer unaware patients (Cohort 2NA) had problems with mobility and self-care (Additional file 1: Appendix Fig. S1). However, moderate or severe pain/discomfort were more prevalent in unaware patients.

Analyses of EQ-5D-5L utility and SDS total scores by clinician/patient-rated severity and patient-rated impact further confirmed that patients with possible TD had worse physical wellness and social functioning than those with no abnormal involuntary movements (Table 3). When adjusted for age, sex, health status, severity of psychiatric condition, functional status, and psychiatric diagnosis, linear regressions based on mean score differences between cohorts indicated that the largest decrements in EQ-5D-5L utility were in Cohort 2A patients with “a lot” of self-reported impact (adjusted mean score difference relative to modified Cohort 1: − 0.121, *P* < 0.001) or “a lot” of self-reported severity (difference: − 0.089, *P* < 0.001). The worst SDS total scores were also found in aware patients who self-reported “a lot” of self-reported impact (adjusted mean score difference: 5.401, *P* < 0.001) or “a lot” of self-reported severity (difference: 3.288, *P* < 0.01).

**Table 3:** EQ-5D-5L and SDS scores by clinician and patient ratings of severity or impact

Severity or impact of possible TD	Cohort 2 or 2A^ab^ versus modified Cohort 1^c^	
	EQ-5D-5L utility score (SE)^d^	SDS total score (SE)^d^
*Cohort 2*^*a*^		
“Some” or “a lot” of clinician-rated severity (n = 204)	− 0.037 (0.015)*	0.267 (0.756)
“A lot” of clinician-rated severity (n = 68)	− 0.044 (0.023)	− 0.732 (1.139)
*Cohort 2A*^*b*^		
“Some” or “a lot” of patient-rated severity (n = 110)	− 0.036 (0.019)	1.428 (0.931)
“A lot” of patient-rated severity (n = 52)	− 0.089 (0.024)***	3.288 (1.243)**
“Some” or “a lot” of patient-rated impact (n = 83)	− 0.042 (0.021)*	1.675 (1.053)
“A lot” of patient-rated impact (n = 33)	− 0.121 (0.031)***	5.401 (1.509)***

*EQ-5D-5L* EuroQoL 5-dimension 5-level questionnaire; *SDS* Sheehan Disability Scale; *SE* standard error; *TD* tardive dyskinesia

*P < 0.05; **P < 0.01; ***P < 0.001 (significantly worse for Cohort 2 subgroup versus modified Cohort 1). Adjusted for age, sex, overall health status, severity of psychiatric condition per clinician impression, functional status of patient per clinician impression, and psychiatric diagnosis

^a^Cohort 2 included all patients with possible TD per clinician assessment. By definition, all Cohort 2 patients had a clinician-rated severity of “some” or “a lot” in ≥ 1 body region (N = 204)

^b^Cohort 2A included patients who were aware of their possible TD. By definition, all Cohort 2A patients rated their severity as “some” or “a lot” in ≥ 1 body region (N = 110). Not all aware patients reported having “some” or “a lot” of impact on daily activities; 27 aware patients either reported “none” for all 7 activities or did not provide a response

^c^Modified Cohort 1 included patients who had no abnormal involuntary movements (N = 450)

^d^Based on linear regression analyses of mean score differences between Cohort 2 subgroups and modified Cohort 1. Negative values for EQ-5D-5L utility score indicate worse health-related quality of life for Cohort 2 populations. Positive values for SDS total scores indicate worse functioning for Cohort 2 populations

### Association between severity/impact of possible TD and EQ-5D-5L utility score

Linear regression analyses conducted in Cohorts 2, 2A, and 2NA indicated no statistical significance for the association between clinician-rated severity and EQ-5D-5L utility in any body region, regardless of whether patients were aware or not aware of their possible TD (Additional file 1: Appendix Table S3).

In contrast, statistical significance was found for the association between patient-rated severity of possible TD and EQ-5D-5L utility in patients who were aware of their possible TD (Cohort 2A) (Table 4). After adjustments for age, sex, health status, severity of psychiatric condition, functional status, and psychiatric diagnosis, the regression coefficient for overall patient-rated severity (summary score based on all body regions) was − 0.028 (*P* < 0.05). Similar coefficients were found for severity in the head/face (− 0.026) and upper extremities (− 0.032), indicating comparable magnitudes of effect on EQ-5D-5L utility; however, these results were not statistically significant. Patient-rated severity in the lower extremities had the largest association with EQ-5D-5L utility, with an adjusted regression coefficient of − 0.060 (*P* < 0.05).

**Table 4:** Association between patient-rated severity/impact of possible TD and EQ-5D-5L/SDS (Cohort 2A)

Severity and impact of possible TD (Cohort 2A)^a^	Mean score (SD)^b^	Regression coefficient (SE)^c^	
		EQ-5D-5L utility	SDS total
*Patient-rated severity*			
Head/face	0.95 (0.75)	− 0.026 (0.026)	2.153 (1.136)
Neck/trunk	0.31 (0.60)	− 0.013 (0.033)	− 1.095 (1.497)
Upper extremities	0.84 (0.75)	− 0.032 (0.026)	1.031 (1.175)
Lower extremities	0.59 (0.72)	− 0.060 (0.027)*	0.539 (1.215)
Summary	2.7 (1.6)	− 0.028 (0.012)*	0.725 (0.560)
*Patient-rated impact*			
Usual activities	0.57 (0.70)	− 0.094 (0.028)***	4.544 (1.228)***
Talking	0.53 (0.70)	− 0.032 (0.027)	1.992 (1.231)
Eating	0.41 (0.65)	− 0.068 (0.030)*	1.987 (1.394)
Breathing	0.11 (0.34)	− 0.062 (0.054)	2.927 (2.511)
Being productive	0.59 (0.69)	− 0.075 (0.028)**	4.031 (1.232)**
Self-care	0.35 (0.60)	− 0.116 (0.032)***	4.759 (1.429)**
Socializing	0.68 (0.74)	− 0.075 (0.026)**	2.818 (1.168)*
Summary	3.2 (3.1)	− 0.023 (0.006)***	1.027 (0.276)***

*EQ-5D-5L* EuroQoL 5-dimension 5-level questionnaire; *SD* standard deviation; *SDS* Sheehan Disability Scale; *SE* standard error; *TD* tardive dyskinesia

*P < 0.05; **P < 0.01; ***P < 0.001 for the linear regression coefficient, indicating that the association was statistically significant. Adjusted for age, sex, overall health status, severity of psychiatric condition per clinician impression, functional status of patient per clinician impression, and psychiatric diagnosis

^a﻿﻿^Cohort 2A included patients who were aware of their possible TD. By definition, all Cohort 2A patients rated their severity as “some” or “a lot” in ≥ 1 body region (N = 110). Not all aware patients reported having “some” or “a lot” of impact on daily activities; 27 aware patients either reported “none” for all 7 activities or did not provide a response

^b^Based on patient ratings of “none” (score = 0), “some” (score = 1), or “a lot” (score = 2), divided by the cohort size (N = 110). For missing values, a score of 0 was assigned. Summary based on summed scores: range, 0 to 8 (severity of possible TD); range, 0 to 14 (impact of possible TD)

^c^Negative regression coefficient indicates an association between higher (worse) severity/impact scores and lower (worse) EQ-5D-5L utility index scores. Positive regression coefficient indicates an association between higher (worse) severity/impact scores and higher (worse) SDS total scores. For these analyses, EQ-5D-5L utility and SDS total scores were the dependent variables. Within each scale, coefficients can be compared to each other for relative strength, but they should not be interpreted as “low” or “high”

Patient-rated impact (not severity) of possible TD had the greatest association with EQ-5D-5L utility (Table 4). After adjustments, regression coefficients (unstandardized) indicated that for Cohort 2A, EQ-5D-5L was significantly associated with the patient-rated impact of TD on 5 activities (self-care, usual activities, being productive, socializing, eating) and on overall patient-reported impact (summary score) (*P* < 0.05 for all coefficients). Since the EQ-5D-5L utility is scored from 0 to 1, and unstandardized coefficients reflect the underlying metric of the scale, the coefficients presented in Table 4 can be interpreted as percentage changes in overall quality of life due to the specific impacts.

### Association between severity/impact of possible TD and SDS total score

Similar to EQ-5D-5L utility, no statistically significant associations were found between clinician-rated severity and SDS total score in any body region (Additional file 1: Appendix Table S3). After adjustments, the regression coefficient for overall patient-rated severity (summary score based on all body regions) was 0.725 (*P* > 0.05) in Cohort 2A, with severity in head/neck having the greatest effect on SDS total score (Table 4). Although coefficients based on patient-rated severity were not statistically significant, the positive coefficients for SDS indicate a positive association with patient-rated severity, suggesting that higher severity was associated with worse disability.

As with EQ-5D-5L utility, patient-rated impact of possible TD had the largest association with SDS total score (Table 4). In Cohort 2A, SDS total score was significantly associated with the patient-rated impact of TD on 4 activities (self-care, usual activities, being productive, socializing) and on overall impact (summary score) (*P* < 0.05 for all coefficients).

## Discussion

In these analyses of RE-KINECT data, which were conducted to address the need for quantitative evaluations of HRQoL in patients with TD, patient-reported outcomes (PROs) regarding health and physical wellness (EQ-5D-5L) and social functioning (SDS) were generally worse in patients with clinician-confirmed possible TD (Cohort 2) than in those with no abnormal involuntary movements (modified Cohort 1). Cohort 2 was characterized by older age, more male patients, a higher prevalence of schizophrenia or schizoaffective disorder, greater severity of psychiatric conditions, worse overall health, and less ability to work/study than modified Cohort 1. After adjusting for these potentially confounding factors, analyses of patient-reported EQ-5D-5L and SDS mean scores indicated that wellness and functioning were most negatively affected in patients who were aware of their possible TD symptoms and rated those symptoms as having “a lot” of severity in ≥ 1 body region or “a lot” of impact on ≥ 1 daily activity. These findings were consistent with results from regression analyses, which showed that patient-reported impact had the largest (and mostly significant) effects on EQ-5D-5L utility and SDS total scores, followed by patient-reported severity. In contrast, no statistically significant associations were found between clinician-rated severity and the PRO measures (EQ-5D-5L utility and SDS total scores).

Among patients who were aware of their possible TD (Cohort 2A), self-reported severity (“some” or “a lot”) was associated with standard PROs (EQ-5D-5L utility, SDS total). These results highlight some important points about awareness and physical/social impact that should be considered when diagnosing and treating patients with TD. Severity terms like “mild” and “severe” (or “some” and “a lot”) are inherently subjective, and patients who are aware of their TD might find even “milder” symptoms to be disruptive, debilitating, or embarrassing. For optimal diagnosis and treatment, it may be important to assess patients’ awareness of their own symptoms and the impact of these symptoms on overall wellness and ability to function—whether self-reported using a formal instrument (EQ-5D-5L, SDS) or simple descriptors (“none”, “some”, or “a lot” of impact). Clinician ratings of TD severity were less likely to be associated with EQ-5D-5L and SDS scores, underscoring the importance of assessing the patient’s perspective.

Assessing the detrimental effects of TD on patients’ physical wellness and social functioning was one of the challenges in this analysis. In a cross-sectional study such as RE-KINECT, assessing causality (i.e., the additive effects of TD) can be hypothesized but not concluded. The analysis might have also been limited by potential overlapping characteristics between modified Cohort 1 (patients with no abnormal involuntary movements) and Cohort 2 (patients with possible TD) due to the fact that patients in both cohorts had been treated with an antipsychotic for ≥ 3 months, primarily for serious mental illnesses such as schizophrenia/schizoaffective disorder, bipolar disorder, and major depressive disorder. Given the functional decrements associated with these conditions [25–27], it was anticipated that both physical and social wellbeing would be diminished in all RE-KINECT patients regardless of cohort. Nevertheless and importantly, EQ-5D-5L and SDS scores were consistently worse in Cohort 2 than in modified Cohort 1 as expected (Tables 2 and 3). However, these differences were not all statistically significant, possibly due to some residual confounding even after adjusting for various patient characteristics (e.g., age, severity of psychiatric condition).

Another objective of this analysis was to explore whether patients who were aware of their possible TD had worse self-reported physical wellness and social functioning than those who were not aware. This appeared to have been the case. Comparisons between Cohort 2A (aware) and Cohort 2NA (not aware) demonstrated similar severity of psychiatric conditions between the cohorts. However, the patients in Cohort 2A (aware) were more likely to self-report poorer overall health (Additional file 1: Appendix Table S1). In addition, Cohort 2A patients had significantly worse SDS scores (total and family/home life), suggesting that patients who are aware of their TD felt a greater negative impact in areas that require interaction with other people. The results also indicate that in patients who are not aware of their TD, it is important to assess the impact of TD on patients’ health using caregiver feedback, including medical issues that the patient might not associate with TD (e.g., pain). Especially in patients with advanced psychotic disorders, family and caregiver accounts of TD movements and impact are valuable in assessing the overall “severity” of the condition.

The emphasis on patients’ experiences in this analysis does not discount the need for regular clinician assessments or caregiver input. Although clinician-rated severity was not significantly associated with EQ-5D-5L or SDS scores, clinician assessment of severity remains a crucial part of the treatment plan for determining the effectiveness of interventions. In patients who are not aware of their symptoms, caregivers can provide information about the location and severity of TD symptoms that are not present during the office visit or are located in areas that are not easily visible, such as the toes. In addition, while patients who reported being unaware of abnormal movements may rate the impacts of TD on functioning as less severe, it remains possible that the abnormal movements could still significantly affect social and occupational relations with others who are distracted by the patient’s movements, regardless of their lack of awareness or denial. Clinician assessments, patient perspectives, and caregiver input should all be considered when evaluating and treating patients with TD.

Limitations for this study have been previously discussed [17]. In brief, it should be noted that RE-KINECT was intended to be a screening study of possible TD with no requirement for “formal” TD diagnoses. Additionally, the cohorts were not matched for any sociodemographic factors, and statistically significant differences between patients with possible TD (Cohort 2) and patients with no abnormal involuntary movements (modified Cohort 1) were found for age, sex, psychiatric conditions, severity of psychiatric conditions, overall health status, and overall functional status (Table 1). However, the adjustments for age, sex, and psychiatric conditions in this analysis should have mitigated some of this variability. It should also be noted that although the EQ-5D-5L and SDS are well-established and standardized instruments (PROs), neither has been specifically validated for TD. However, the strong associations found between EQ-5D-5L/SDS and patient-reported impact of possible TD indicate that both scales may be appropriate for assessing HRQoL in patients with TD. Finally, the ordinal values assigned to various outcomes for the regression analyses (e.g., 0 = "none", 1 = "some", 2 = "a lot") assumes that the qualitative differences between responses are similar, which may or may not be true. The rationale for this approach was to provide a relatively simple method that can be easily reproduced. The findings based on this approach were sufficient to confirm the applicability of a simple impact assessment (“none”, “some”, or “a lot”) to real-world practice.

## Conclusions

The results of this analysis indicate that physical wellness and social functioning were diminished in patients with possible TD, particularly in those who were aware of their abnormal involuntary movements and rated those movements as having “a lot” of impact on daily activities. Patient-rated severity and impact of possible TD, but not clinician-rated severity, were significantly associated with EQ-5D-5L utility or SDS total scores. These outcomes suggest that in addition to assessing the presence and severity of patients’ abnormal movements during usual care visits, clinicians or their staff may need to ask patients about how TD adversely affects their HRQoL (particularly as it relates to their daily activities) and consider these impacts when making and evaluating treatment plans. For some patients, such questions may be as important (or possibly even more important) than symptom severity. For patients not aware of their TD, discussion with caregivers may be needed to determine the impact of TD on patients’ health and daily activities. Such discussions might be facilitated by specific and descriptive questions that are specifically focused on social withdrawal (avoiding social interaction or events, isolation, embarrassment) or physical disabilities associated with TD symptoms (difficulty swallowing, breathing, walking, or writing).

## Supplementary Information

**Additional file 1**. Appendix tables and figures.

## Data Availability

The datasets used and/or analyzed during the current study are available from the study sponsor (Neurocrine Biosciences, Inc.) upon reasonable request for research purposes.
