# Supplementary material for: Impact of possible tardive dyskinesia on physical wellness and social functioning: results from the real-world RE-KINECT study
Source: J Patient Rep Outcomes. 2023 Mar 9;7:21. doi: 10.1186/s41687-023-00551-5 (PMC9998754; doi:10.1186/s41687-023-00551-5)
Supplement: Supplementary file 1 — Additional file 1. Appendix tables and figures. [file 41687_2023_551_MOESM1_ESM.pdf]

APPENDIX

In RE-KINECT, 204 patients who had abnormal involuntary movements that were confirmed by a clinician as possible tardive dyskinesia (TD) were assigned to Cohort 2. Within this cohort, 110 self-reported having abnormal involuntary movements within the past 4 weeks and were therefore considered "aware" of their possible TD **(Cohort 2A)**. Data for the remaining 94 patients who were "not aware" of their possible TD **(Cohort 2NA)** are presented in this appendix, with comparisons to Cohort 2A.

Table 1. Demographics and Clinical Characteristics

	Cohort 2A (N=110)	Cohort 2NA (N=94)	*P*-Value[a]
Age, mean (SD)	54.8 (13.1)	54.5 (14.2)	0.877
Sex, n (%)			
Male	54 (49.1)	46 (48.9)	0.983
Female	56 (50.9)	48 (51.1)	
Psychiatric condition, n (%)[b]			
Schizophrenia or schizoaffective disorder	56 (51.0)	51 (54.3)	0.635
Mood disorder or other psychiatric disorder[c]	68 (61.8)	66 (70.2)	0.210
Severity of psychiatric condition, n (%)[d]			
Normal, not ill	4 (3.6)	3 (3.2)	
Minimally ill	11 (10.0)	16 (17.0)	
Mildly ill	36 (32.7)	32 (34.0)	
Moderately ill	40 (36.4)	27 (28.7)	0.423
Markedly ill	15 (13.6)	11 (11.7)	
Severely ill	4 (3.6)	5 (3.3)	
Among the most severely ill	0	0	
Overall health status, mean (SD)[e]	5.1 (2.7)	4.3 (2.8)	0.029
Overall functional status, n (%)[d]			
Working/studying independently	41 (37.3)	25 (26.6)	
Working/studying with assistance	21 (19.1)	23 (24.5)	0.195
Not working/studying	48 (43.6)	46 (48.9)	

[a] *P*-value for Cohort 2NA (not aware of possible TD) versus Cohort 2A (aware of possible TD). For questions or items that allowed more than 1 response (i.e., categories not mutually exclusive), *P*-values are provided for each response. Chi-squared tests were used for categorical variables; t-tests were used for continuous variables.

[b] Based on questionnaire responses (i.e., not diagnostic medical codes).

[c] Includes anxiety disorder or symptoms, bipolar disorder, major depressive disorder, post-traumatic stress disorder, personality disorder, attention deficit hyperactivity disorder, substance use disorder, and other psychotic disorders.

[d] Per clinician impression.

[e] Per patient self-report; higher scores indicate worse overall health.

Table 2. Mean EQ-5D-5L and SDS Scores

	Cohort 2A		Cohort 2NA		
	n	Mean (SD)	n	Mean (SD)	*P*-Value[a]
EQ-5D-5L scores[b]					
Health state VAS score	110	65.4 (24.2)	94	68.4 (26.2)	0.757
Utility index score	105	0.71 (0.21)	92	0.72 (0.21)	0.720
SDS scores[c]					
Work/school	71	4.5 (3.3)	40	3.6 (3.4)	0.428
Social life	109	4.5 (3.2)	94	3.4 (3.5)	0.194
Family/home life	109	4.4 (3.2)	94	3.0 (3.2)	0.030
Total score	109	13.5 (9.1)	94	9.7 (9.1)	0.045

[a] *P*-value for Cohort 2NA (not aware of possible TD) versus Cohort 2A (aware of possible TD). Adjusted for age, sex, overall health status, severity of psychiatric condition per clinician impression, functional status of patient per clinician impression, and psychiatric diagnosis.

[b] VAS score range: 0 (worst possible health) to 100 (best possible health). Utility index score range: 0 (health condition equivalent to death) to 1 (perfect health).

[c] Dimension score range: 0 (no impairment) to 10 (highly impaired). Total score range: 0 to 30 (sum of dimension scores). Total score was calculated for patients who had ≥2 domain scores. When only 1 domain was missing, the average of the patient's observed score was imputed.

EQ-5D-5L, EuroQol 5-Dimension 5-Level questionnaire; SD, standard deviation; SDS, Sheehan Disability Scale; TD, tardive dyskinesia; VAS, visual analog scale.

Figure 1. Distribution of EQ-5D-5L Scores

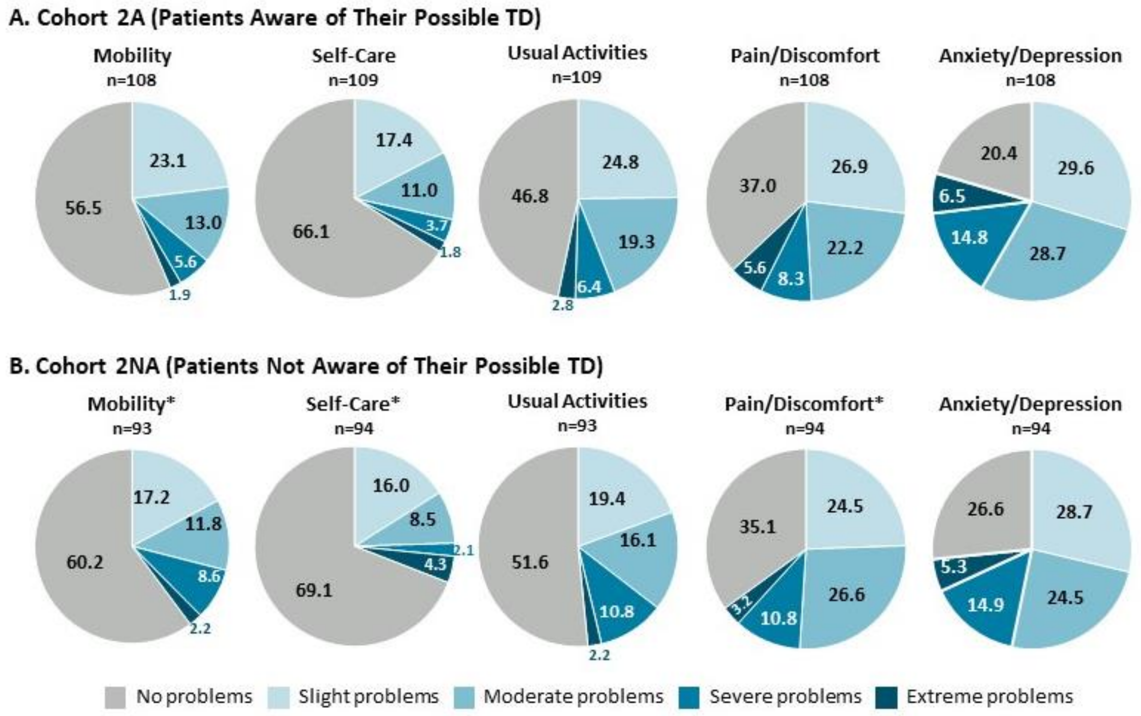

*P<0.05 vs. Cohort 2A.

EQ-5D-5L, EuroQoL 5-Dimension 5-Level questionnaire; TD, tardive dyskinesia.

Table 3. Association Between Severity of Possible TD and EQ-5D-5L Utility Score and SDS Total Score

Clinician-Rated Severity, Cohort 2 Patients[a]	Mean Score (SD)[b]	Regression Coefficient (SE)[c]	
		EQ-5D-5L Utility	SDS Total
Cohort 2 (N=204)			
Head/face	0.86 (0.72)	0.001 (0.019)	0.196 (0.879)
Neck/trunk	0.25 (0.53)	0.014 (0.026)	-0.868 (1.182)
Upper extremities	0.69 (0.65)	-0.007 (0.021)	0.684 (0.970)
Lower extremities	0.50 (0.64)	-0.039 (0.021)	0.242 (0.803)
Summary	2.3 (1.4)	-0.007 (0.010)	0.027 (0.440)
Cohort 2A (N=110)			
Head/face	0.83 (0.75)	0.008 (0.026)	0.496 (1.182)
Neck/trunk	0.26 (0.55)	-0.011 (0.037)	-0.280 (1.683)
Upper extremities	0.74 (0.66)	-0.032 (0.029)	1.455 (1.310)
Lower extremities	0.56 (0.68)	-0.037 (0.028)	-0.038 (1.282)
Summary	2.4 (1.5)	-0.015 (0.013)	0.354 (0.608)
Cohort 2NA (N=94)			
Head/face	0.90 (0.69)	0.017 (0.031)	-0.430 (1.336)
Neck/trunk	0.25 (0.51)	0.075 (0.040)	-0.058 (1.751)
Upper extremities	0.65 (0.64)	0.016 (0.031)	-0.418 (1.450)
Lower extremities	0.43 (0.58)	-0.035 (0.034)	0.464 (1.501)
Summary	2.2 (1.4)	0.012 (0.015)	-0.277 (0.640)

[a] Cohort 2 includes all patients with possible TD per clinician assessment. Cohort 2A represents patients who were aware of having possible TD within the past 4 weeks. Cohort 2NA represents patients who were not aware of having possible TD movements within the past 4 weeks.

[b] Based on clinician ratings of "none" (score=0), "some" (score=1), or "a lot" (score=2). For missing values, a score of 0 was assigned. Summary based on summed scores (range, 0 to 8).

[c] Negative regression coefficients indicate inverse relationships between higher (worse) severity/impact scores and lower (worse) EQ-5D-5L utility index scores. Positive regression coefficients indicate direct relationships between higher (worse) severity/impact scores and higher (worse) SDS total scores. For these analyses, EQ-5D-5L utility and SDS total scores were the dependent variables. Within each scale, coefficients can be compared to each other for relative strength, but they should not be interpreted as "low" or "high". No statistically significant coefficients were found.

EQ-5D-5L, EuroQoL 5-Dimension 5-Level questionnaire; SD, standard deviation; SDS, Sheehan Disability Scale; SE, standard error; TD, tardive dyskinesia.

List of Study Sites

For all RE-KINECT study sites, which are listed below, protocol and ethics approvals were provided by Quorum Review IRB (Seattle, WA).

- Premier Psychiatric Research Institute, LLC (Lincoln, NE)
- Arkansas Psychiatric Clinic (Little Rock, AR)
- Sarkis Clinical Trials (Gainesville, FL)
- Pact Atlanta, LLC (Decatur, GA)
- Red River Medical Research Center (Oklahoma City, OK)
- Cherry Street Services (Grand Rapids, MI)
- Finger Lakes Clinical Research (Rochester, NY)
- Galiz Research (Hialeah, FL)
- University of South Florida (Tampa, FL)
- Augusta University (Augusta, GA)
- Red Oak Psychiatry Associates (Houston, TX)
- Clinical Trials of America–NC (Hickory, NC)
- Nasr Psychiatric Services (Michigan City, IN)
- AMR Conventions Research (Naperville, IL)
- Healthy Perspective (Nashua, NH)
- Rochester Center for Behavioral Medicine (Rochester, MI)
- Valden Medical LLC (Honolulu, HI)
- Florida International Research Center (Miami, FL)
- Ocean Blue Medical Research Center (Miami Springs, FL)
- Carolina Outreach (Durham, NC)
- John Hardy, MD, PC (Pueblo, CO)
- APG Research, LLC (Orlando, FL)
- Psychiatric Behavior Solutions (Salt Lake City, UT)
- Advanced Research Center (Anaheim, CA)
- California Pharmaceutical Research Institute (Anaheim, CA)
- Psych Care Consultants Research (St. Louis, MO)
- Baylor College of Medicine (Houston, TX)
- Texas Tech University Health Sciences (San Antonio, TX)
- Elite Medical Wellness (Lake Charles, LA)
- Icahn School of Medicine at Mount Sinai (New York, NY)
- Tuscaloosa VA Medical Center (Tuscaloosa, AL)
- Clinical Training and Research Institute (Burlingame, CA)
- Manoj V Waikar, MD (Los Gatos, CA)
- University of Missouri Kansas City (Kansas City, MO)
- Prisat PA (Jacksonville, FL)
- Jamaica Hospital Medical Center (Jamaica, NY)
- Beacon Medical Group Clinical Research (South Bend, IN)
